# Genetic and expression studies of *SMN2 *gene in Russian patients with spinal muscular atrophy type II and III

**DOI:** 10.1186/1471-2350-12-96

**Published:** 2011-07-15

**Authors:** Galina Yu Zheleznyakova, Anton V Kiselev, Viktor G Vakharlovsky, Mathias Rask-Andersen, Rohit Chavan, Anna A Egorova, Helgi B Schiöth, Vladislav S Baranov

**Affiliations:** 1 Laboratory for Prenatal Diagnostics of Inherited Diseases, Ott's Institute of Obstetrics and Gynecology RAMS, Mendeleevskaya line 3, 199034, Saint-Petersburg, Russia; 2 Department of Biochemistry, Faculty of Biology and Soil Science, Saint-Petersburg State University, Universitetskaya emb. 7/9, 199034, Saint-Petersburg, Russia; 3 Unit of Functional Pharmacology, Department of Neuroscience, Uppsala University, Box 593, Husargatan 3, 75124 Uppsala, Sweden

## Abstract

**Background:**

Spinal muscular atrophy (SMA type I, II and III) is an autosomal recessive neuromuscular disorder caused by mutations in the survival motor neuron gene (*SMN1*). *SMN2 *is a centromeric copy gene that has been characterized as a major modifier of SMA severity. SMA type I patients have one or two *SMN2 *copies while most SMA type II patients carry three *SMN2 *copies and SMA III patients have three or four *SMN2 *copies. The *SMN1 *gene produces a full-length transcript (FL-SMN) while *SMN2 *is only able to produce a small portion of the FL-SMN because of a splice mutation which results in the production of abnormal SMNΔ7 mRNA.

**Methods:**

In this study we performed quantification of the *SMN2 *gene copy number in Russian patients affected by SMA type II and III (42 and 19 patients, respectively) by means of real-time PCR. Moreover, we present two families consisting of asymptomatic carriers of a homozygous absence of the *SMN1 *gene. We also developed a novel RT-qPCR-based assay to determine the FL-SMN/SMNΔ7 mRNA ratio as SMA biomarker.

**Results:**

Comparison of the *SMN2 *copy number and clinical features revealed a significant correlation between mild clinical phenotype (SMA type III) and presence of four copies of the *SMN2 *gene. In both asymptomatic cases we found an increased number of *SMN2 *copies in the healthy carriers and a biallelic *SMN1 *absence. Furthermore, the novel assay revealed a difference between SMA patients and healthy controls.

**Conclusions:**

We suggest that the *SMN2 *gene copy quantification in SMA patients could be used as a prognostic tool for discrimination between the SMA type II and SMA type III diagnoses, whereas the FL-SMN/SMNΔ7 mRNA ratio could be a useful biomarker for detecting changes during SMA pharmacotherapy.

## Background

Spinal muscular atrophy (SMA) is an autosomal recessive neuromuscular disorder characterized by degeneration of alpha motor neurons in the anterior horns of the spinal cord. SMA is one of the leading genetic causes of infant mortality, with an incidence of about 1 in 8000 live births and with a carrier frequency of about 1 in 40 [1]. SMA subdivides into types I, II, and III based on age of onset and clinical severity [2], and are all caused by mutations within the survival motor neuron gene (*SMN1*) located on chromosome 5q13. Approximately 95 percent of these patients have a homozygous deletion of exon 7 in the *SMN1 *gene. Small intragenic mutations have been found in the remaining affected individuals who do not lack both copies of *SMN1 *[3]. *SMN2 *is an almost identical centromeric *SMN1 *copy, with 8 nucleotide substitutions: five in the introns and three in exons 6, 7 and 8 [4]. Single-nucleotide transition of C to T at position 6 of *SMN2 *exon 7 does not change an amino acid, but causes disruption of a splicing enchancer site, which results in mRNA lacking exon 7 (SMNΔ7) [5]. Most of the *SMN1 *transcripts are full-length whereas the majority of the *SMN2 *transcripts lack exon 7. Consequently, *SMN2 *is not able to compensate for the loss of exon 7 in *SMN1 *of the SMA patients. Nevertheless, the *SMN2 *copy number is considered to be a modifying factor for the clinical severity of SMA. SMA type I patients have one or two *SMN2 *copies while most SMA type II patients carry three *SMN2 *copies and SMA III patients have three or four *SMN2 *copies [6-8].

The *SMN1 *gene normally produces full-length SMN mRNA, whereas conversely approximately only 20 percent of the full-length SMN mRNA is produced from the *SMN2 *gene [9]. This observation suggests a potential significant distinction in the FL-SMN transcript levels among healthy individuals, carriers and different types of SMA patients. However, there are conflicting results concerning the differences between the levels of FL-SMN and Δ7SMN mRNA in SMA patients, carriers and healthy people [10-12]. Decreased FL-SMN expression has been shown only for type I SMA patients. Fluctuations in transcript levels throughout drug treatment were also observed in recent clinical trials [13]. However, it remains unclear whether the levels of FL-SMN and Δ7SMN mRNA are associated with the type of disease or the copy number of *SMN2*.

In this study, we performed *SMN2 *gene copy number determination for patients affected by SMA type II and III (42 and 19 patients, respectively). We also present two case-reports: one SMA type III patient and his asymptomatic sister, and one SMA type II patient and his asymptomatic mother being homozygous for the absence of the *SMN1 *gene. To evaluate the importance of the FL-SMN/SMNΔ7 mRNA ratio as a SMA biomarker, we carried out RT-qPCR based quantification of FL-SMN and SMNΔ7 mRNA levels in a small group of type II and III SMA patients.

## Methods

### Patients

Genetic analysis was approved by the ethics committee at Ott's Institute of Obstetrics and Gynecology RAMS. The adult patients and parents of all children gave written informed consent to the diagnostic procedures. Molecular testing for deletion in the *SMN1 *gene was performed in 190 Russian families from the North-Western region of Russia with SMA including 172 affected individuals, 243 parents, 14 siblings and 44 other relatives (totally 301 relatives were tested). *SMN2 *gene copy number determination was performed for 42 type II SMA-patients and 19 type III SMA-patients. *SMN2 *gene copy number was also determined in several clinically healthy family members including four parents, two siblings and one grandmother. Blood samples for the determination of *SMN *gene expression were obtained from five SMA patients, one SMA carrier and two healthy individuals (Table 1).

**Table 1:** FL-SMN and Δ7 SMN baseline transcript measurement in SMA patients, SMA carrier and control individuals

	Sex	SMA type	Number of SMN2 copies	Relative level of FL-SMN transcript	Relative level of Δ7 SMN transcript	FL-SMN/Δ7SMN ratio
1	M	III	3	2.6	2.11	1.23

2	M	II	3	0.85	1.24	0.69

3	F	II	3	1.74	0.83	2.09

4	F	III	4	3.23	2.78	1.16

5	F	asymptomatic	4	1.39	2.48	0.56

	F	carrier	4	1.40	2.02	0.69

	F	control	2	3.25	1.05	3.10

	M	control	1	3.87	0.53	7.3

### DNA isolation

Genomic DNA was extracted from peripheral blood leukocytes of SMA patients, their relatives and healthy individuals using the phenol-chloroform method [14].

### Polymerase chain reaction - restriction fragment length polymorphism (PCR-RFLP) and single strand conformation analysis (SSCA)

All SMA patients and their relatives were tested for *SMN1 *exon 7 and 8 deletions by means of SSCA (for exon 7) and PCR-RFLP analysis (for exon 8). PCR-RFLP and SSCA methods were performed as described previously [15].

### Real-time PCR for *SMN2 *gene copy number determination

We applied the TaqMan real-time PCR method as previously described [16]. The DNA sample of the SMA patient with four *SMN2 *gene copies was used as standard in all amplification reactions. The presence of four copies was established earlier using reference DNA samples kindly provided by prof. B. Wirth (Institute of Human Genetics, University of Cologne). In each run we also used DNA samples from SMA patients with two and three copies as additional standards. The β-globine (*HBB*) gene was used as a reference locus in line with previously published primers and probe [17].

PCR was performed using the thermocycler Rotor-Gene 3000 (Corbett Life Science, Australia). PCR was carried out in a total volume of 20 μl and contained 10-160 ng of genomic DNA, 1 μl of each primers (1 A_260 _o.u.) (Syntol, Russia), 2 μl of 2 μM *SMN2 *MGB (Minor Groove Binder) probe (Applied Biosystems, USA), 2 μl of 2 μM of HBB probe (Syntol, Russia), 2.5 μl of 2 mM dNTP mix (Fermentas, Lithuania), 2 μl of 25 mM MgCl_2_, 2 μl of 10× polymerase buffer and 0.3 μl of Taq DNA polymerase (Sileks, Russia).

We amplified 160 ng, 80 ng, 40 ng, 10 ng of standard DNA and 50 ng of each tested DNA in one run. PCR conditions were as follows: 94°C for 10 minutes, 40 cycles consisting of 95°C for 20 seconds, 60°C for 1 minute. Data was analyzed by Rotor-Gene version 6.1.71 software. All samples were measured three times and a final result was inferred by averaging data. The value ranges for two, three and four *SMN2 *gene copy number were estimated. Confidence of differences between the values range was confirmed using unpaired Student's t-test (p < 0.01), using GraphPad Prism v5.03 (GraphPad, USA).

### RNA isolation and cDNA synthesis

Peripheral blood was collected into Vacuette tubes (Greiner Bio One, USA). 2 ml of blood was lysed with hemolysis buffer (8.4 g/l NH_4_Cl) for 30 minutes. The leukocyte phase was obtained by centrifuging the blood sample at 1500 rpm for 20 minutes. Collected cells were stored in RNAlater solution (Quiagen, Germany) for up to twelve months at -80°C. RNA isolation was performed simultaneously from all samples using RiboPure - Blood Kit (Applied Biosystems). For cDNA synthesis 12 μl of each RNA sample was incubated with 0.5 μl of 25 mM dNTP mix (Fermentas) and 0.5 μl of hexaprimers (50 A_260 _o.e.) (Roche) at 65°C for 5 minutes. Samples were kept on ice for 1 minute. 4 μl of 5× FS buffer, 2 μl 0, 1 M DTT and 1 μl of M-MLV Reverse transcriptase (Invitrogen) were added to samples. Reactions were incubated at 25°C for 10 minutes, then at 37°C for 1 hour, then at 95°C for 15 minutes.

### Quantitative reverse transcriptase real-time PCR

The assay was carried out on a MyiQ thermal cycler (Bio-Rad Laboratories, Sweden), using 96-well plates. The primers were designed using Beacon Primer Design 4.0 software (Premier Biosoft, USA). Primers for full-length SMN transcripts amplification were forward 5'-CTGATGCTTTGGGAAGTAT-3', reverse 5'-GCCAGCATTTCTCCTTAA-3', for Δ7SMN transcripts amplification were forward 5'-GTCCAGATTCTCTTGATGAT-3', reverse 5'-GCCAGCATTTCCATATAATAG-3'. We also amplified two reference genes - glyceraldehyde-3-phosphate dehydrogenase (*GAPDH*) and histone *H3b*. Primers sequences were following: for *GAPDH *locus forward 5'-CGCCAGCCGAGCCACATC-3', reverse 5'-CGCCCAATACGACCAAATCCG-3'; for *H3b *locus forward 5'-ATCCGCCGCTACCAAAAG-3', reverse 5'-CGAAGATCGGTCTTGAAGTC-3'. Reactions were performed in the final volume of 20 μl and contained 5 μl of cDNA (5 ng/μl), 0.05 μl of each primer (100 pmol/μl), 1 μl DMSO, 0.5 μl of SYBR GREEN I (1:50000; Invitrogen, Sweden) in TE buffer (pH 7.8), 0.2 μl of 25 mM dNTP mix (Fermentas), 2 μl 10× buffer, 1.6 μl of 50 mM MgCl_2_, 0.08 μl of Taq DNA polymerase (Biotools, Spain). Cycling conditions were as follows: 3-minutes initial denaturation step, followed by 40 cycles of 95°C for 20 seconds, 20 seconds at 55-60.5 (optimal annealing temperature of primers), 30 seconds at 72°C. Fluorescence was measured after the elongation phase. 81 cycles of 10 sec at 55°C with increasing increments of 0.5°C per cycle was performed for melting curve analysis. A negative control for each primer's pair was included on each plate. All samples were run in duplicate. MyiQ software v 1.04 (Bio-Rad Laboratories, Sweden) was used to process real-time PCR data and determine threshold cycle (Ct) values. Melting curve analysis was performed to confirm that only one product was amplified and there were not any products in negative controls. LinRegPCR was used to calculate PCR efficiencies for each sample. Outliers were excluded and the average PCR efficiency for each primers pair was calculated using Grubbs' test for outliers (GraphPad, USA). Relative quantities with standard deviations were calculated using the delta Ct method. Normalization was performed via the GeNorm method using expression levels of GAPDH and H3b.

## Results

### Quantification of *SMN2 *gene copy number in patients with spinal muscular atrophy

In this study we performed *SMN2 *dosage analysis in 42 type II-, and 19 type III SMA patients. The *SMN2 *gene copy number was determined using a real-time PCR quantification method with MGB-TaqMan probes [16]. DNA samples from the SMA patients carrying between one and four *SMN2 *copies were used as controls for the analysis.

We found that the sample-to-sample variation after relative quantification analysis for the two *SMN2 *gene copies per genome was in the range of 0.73-1.32; for three copies per genome it was in range of 1.27-1.72; and for four copies per genome it was in range of 1.71-2.2. These values were multiplied by a factor of two to obtain the gene copy number. These results are in agreement with previously published data obtained by real-time PCR approaches using TaqMan or LightCycler techniques [8,16,18].

**Figure 1:**
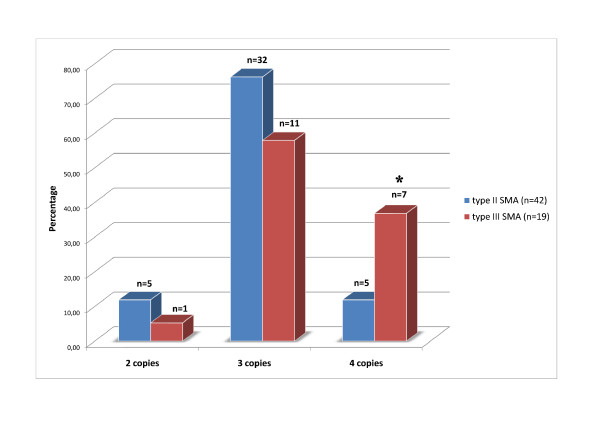
**Frequencies (%) of the *SMN2 *gene copy numbers in patients with SMA type II and III**. * - χ2-test was used for the comparison of *SMN2 *gene copy number between type II and type III patients (p = 0.0233, χ2 = 5.148).

The results of the *SMN2 *gene copy number quantification in the SMA patients are presented in Figure 1. It can be seen that most of the SMA type II patients have three copies of *SMN2 *gene per genome (76.2%), whereas two and four copies were found in equal numbers of the patients (11.9%). For the SMA type III group, we found three copies of *SMN2 *gene for lesser number of patients (57.9%) while four copies were found in 7 of 19 patients (36.8%). It should be noted that we found one patient affected by SMA type III bearing two copies of the *SMN2 *gene (5.3%). The average *SMN2 *copy number in type II and type III patients was found to be similar (3.0 ± 0.49 vs 3.32 ± 0.58; p > 0.01). We were not able to reveal significant differences between the SMA type II and III patients bearing two copies of the *SMN2 *gene (5 of 42 type II patients and one of 19 type III patients) (p > 0.05). This was likely due to the low number of individuals in each group. The difference in frequency of three copies of *SMN2 *gene between type II and III patients was also not found to be significant (32 of 42 type II patients and 11 of 19 type III patients) (p > 0.05). However, we did find a significant difference in the frequency of having four *SMN2 *gene copies between type II and type III patients (p = 0.0233, χ2 = 5.148).

### Asymptomatic family members lacking both copies of the *SMN1 *gene

We carried out routine molecular genetic diagnoses in 301 clinically healthy members of Russian SMA families, including obligatory deletion carriers such as parents and siblings who have a chance of being heterozygous carriers, and other relatives. In each family, deletions of exons 7 and 8 of *SMN1 *and *SMN2 *were determined by means of SSCP analysis and RFLP analysis [15]. As a result of the diagnosis, we revealed two families of asymptomatic individuals who were homozygous for the absence of exons 7 and 8 of *SMN1*. Therefore, the prevalence of asymptomatic cases in Russian families may be estimated at approximately 0.7% of first-degree relatives of SMA patients.

#### Family 1 (no. S265)

**Figure 2:**
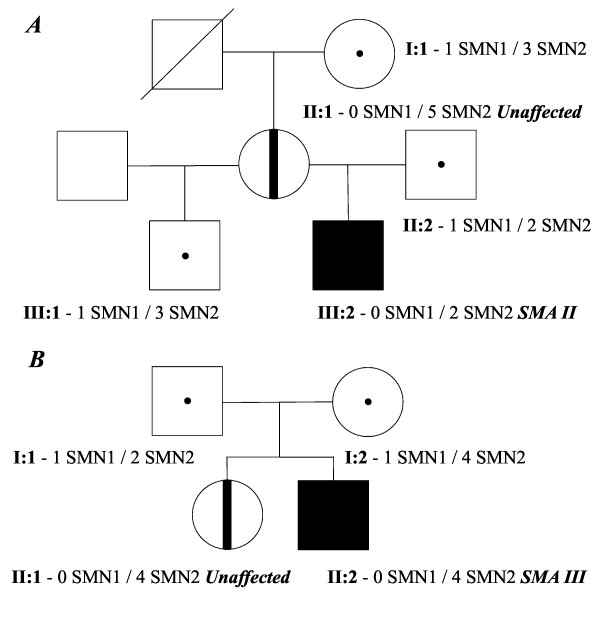
**Pedigrees of spinal muscular atrophy (SMA) families with biallelic absence of the SMN1 gene in unaffected subjects**. (a) family 1, (b) family 2; circles represent females, squares represent males, black symbols represent biallelic SMN1 absence in affected individuals, dot symbols represent heterozygous SMA carriers, vertical line symbols represent biallelic SMN1 absence in unaffected individuals.

There were two half-siblings in family 1 (Figure 2a). The younger brother was affected with SMA type II. The diagnosis was confirmed by molecular testing of deletions of the *SMN1 *exon 7 and 8. Molecular diagnostics showed a heterozygous deletion carrier state in a healthy stepbrother (III:1). The father (II:2) of the affected boy (III:2) was found to be heterozygous for the deletion, whereas the 45 year old mother (II:1) carried a biallelic deletion of exon 7 and 8 of *SMN1*. Quantification of *SMN2 *gene copy number in the members of family 1 allowed us to explain the absence of SMA symptoms in the mother. It was shown that the father and the SMA type II affected son carried two copies of *SMN2*. Three copies of *SMN2 *were found in the healthy stepbrother. Furthermore, five copies of the *SMN2 *gene were found in the asymptomatic mother.

#### Family 2 (no. S280)

There were two siblings in family 2 (Figure 2b). The younger brother was affected with SMA type IIIb. The actual SMA symptoms debuted at age 21. Molecular diagnostics confirmed the original diagnosis and revealed a homozygous deletion of exon 7 and 8 of *SMN1*. The same analysis showed biallelic deletion in a healthy 25 years old sister (II:1). Both parents were found to be deletion carriers. The number of *SMN2 *gene copies was determined in the members of family 2. We found two copies of *SMN2 *in the father and four copies in the mother. The analysis revealed four copies of *SMN2 *gene in the proband and his asymptomatic sister.

### Measurement of SMN baseline expression levels

In the present study we have developed a novel assay based on real-time PCR, which allows the quantification of FL-SMN and SMNΔ7 transcripts. The assay was evaluated by characterization of the *SMN *baseline expression level in blood samples taken from eight individuals - two patients affected by SMA type II, two patients affected by SMA type III, one asymptomatic individual, one SMA carrier and two healthy controls (Table 1). The assay was used to investigate whether FL-SMN mRNA levels are reduced in the patients compared with healthy individuals.

**Figure 3:**
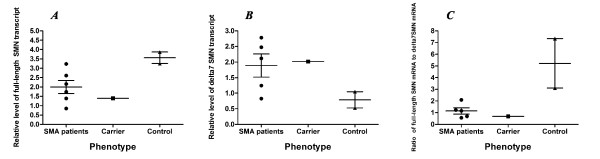
**SMN gene baseline expression variability in SMA patients and healthy individuals**. (a) the variability of baseline full-length SMN transcript level; (b) the variability of baseline SMNΔ7 transcript level; (c) the variability of baseline FL-SMN/SMNΔ7 mRNA ratio.

Baseline level of the FL-SMN mRNA expression varied greatly in the SMA patients in range 0.85 - 3.23 (Figure 3a). Mean ± SEM was found to be 1.97 ± 0.43. In addition, sustained variability was observed in the baseline level of SMNΔ7 mRNA expression in range 0.83 - 2.78. Mean ± SEM was found to be 1.89 ± 0.37 (Figure 3b). Thus the mRNA levels of FL-SMN and SMNΔ7 in the control subjects overlapped with the values for the SMA patients, which represented similar, slightly lower (for FL-SMN) or slightly higher (for SMNΔ7) values. It is suggested that the similarity in expression levels between control individuals and the patients may be due to increased *SMN2 *gene copy number. Three SMA patients from the studied cohort were found to have three *SMN2 *gene copies whereas the other two had four *SMN2 *gene copies (Table 1).

Previously it has been shown that FL-SMN/SMNΔ7 mRNA ratio can be used in the SMN expression analysis in order to avoid confounding effects of FL-SMN and SMNΔ7 mRNA level fluctuations [13]. Taking this approach we analysed FL-SMN/SMNΔ7 mRNA ratio in the patients and control individuals. It can be seen in Figure 3c that the quantification of FL-SMN/SMNΔ7 mRNA ratio revealed a difference between SMA patients and healthy controls. This parameter varied in the range of 0.56 - 2.10. The mean ± SEM was found to be 1.15 ± 0.27.

## Discussion

By comparing the *SMN2 *gene copy number and clinical features of patients with SMA, we have revealed, for the first time in a Russian population, a significant correlation between mild clinical phenotype (SMA type III) and a presence of four copies of *SMN2 *gene. The *SMN2 *gene is considered to be a major modifier of SMA. Despite the presence of the splice mutation in exon 7, the *SMN2 *gene is able to produce up to 10% of FL-SMN mRNA. Multiplication of *SMN2 *gene copies in the genome of SMA patients can therefore lead to variability in the SMA phenotype [8,19,20]. Quantification of the *SMN2 *gene copy number has been suggested to be an important prognostic criterion for SMA diagnosis [8]. In our study, we suggest that the SMA patients bearing four copies of the *SMN2 *gene can be considered as type III patients rather than the II type. This is because, according to the data obtained (Figure 1) 12 percent of SMA type II patients were found to have four copies. Thus, the specificity of the *SMN2 *gene copy number as a prognostic tool can be estimated as an 88 percent risk for a child with four *SMN2 *gene copies to have SMA type III. For example in a previous study, the risk of developing SMA type III in a child with four *SMN2 *copies was estimated to be 83.6 percent [8]. This finding is in concordance with previously published data concerning genotype-phenotype correlation in SMA patients [8,19]. However, it should be noted that this genotype-phenotype correlation is not absolute and can be influenced by modifying genes, point mutations or conversions of *SMN2 *[8]. Our results suggest that the *SMN2 *gene copy measurements in SMA patients can be used as a prognostic tool for discrimination between the SMA type II and SMA type III diagnosis.

The importance of the *SMN2 *gene copy number determination can be further illustrated by our findings in families that have unaffected relatives with a homozygous loss of the *SMN1 *gene. Being an exceptionally rare event, homozygous deletion of *SMN1 *exon 7 and 8 in unaffected relatives of SMA patients has been reported several times in American, German and Polish SMA families [21-24]. In this study we were able, for the first time, to report two cases of asymptomatic individuals from Russian SMA families. In both of the cases of asymptomatic loss of the *SMN1 *gene the influence of *SMN2 *copy number on the SMA severity was demonstrated. In family 1, we see that the higher *SMN2 *copy number completely abolishes the SMA phenotype in a mother of a child severely affected by SMA type II. The second case demonstrates that four copies of *SMN2 *results in a very mild SMA phenotype in a man homozygous for absence of *SMN1*. Conversely, no symptoms were observed in the sister who had the same genotype. This can only be explained by influence on SMA phenotype by genetic factors other than the *SMN2 *gene copy number. Family 2 represents a rare case of siblings with identical *SMN1 *mutations, identical *SMN2 *copy number, but discrepancy in phenotype. This finding suggests that there are additional factors, such as mutations, genetic and epigenetic modifiers to influence the severity of SMA. For instance, higher expression of plastin 3 has been observed in asymptomatic *SMN1*-deleted females, which has been shown to rescue axon growth defects in animal models for *SMN1 *deficiency [25]. The c.859G > C substitution in *SMN2 *gene has also been suggested as a positive modifier of SMA phenotype [26]. This substitution forms a new exonic splicing enhancer that increases the amount of full-length *SMN2 *mRNA. The c.859G > C variant was detected in SMA patients discordant for the disease severity and the *SMN2 *gene copy number [27]. Variations in the disease progression between SMA patients with identical number of *SMN2 *gene can also be explained by differences in DNA methylation. Methylation of a small part of CpG islands in the region of upstream and downstream of the translational *SMN2 *start site has been identified to correlate with the disease severity [28]. Gender could be suggested as SMA severity modifiyng factor; to date most of asymptomatic persons with homozygous loss of the *SMN1 *gene are females [29].

The number of *SMN2 *gene copies in SMA patients has been studied intensively in several previous studies including different populations. However, to date no previous publications concerning Russian populations have been analysed. Here we present *SMN2 *dosage analysis in 61 SMA patients who originated from North-Western Russia. Individuals affected by SMA type II and III were subjected to the analysis because it has been suggested that their milder phenotype is a result of an increase of the *SMN2 *gene copy number. The distribution of *SMN2 *gene copy number observed in the SMA patients from the northwestern region of Russia was compared with the distributions reported for the SMA patients from some other populations. We did not observe any difference in *SMN2 *gene copy number distribution between Russian and German, Spanish and Vietnamese SMA patients [8,18,19].

Recent clinical trials on SMA treatment by histone deacetylation inhibitors such as valproic acid and phenylbutyrate have revealed some promising results. These trials focused on children with SMA types II and III [13,30-32]. Our clinical experience, from a study of how valproic acid can influence the symptoms of SMA, highlights the necessity to elaborate with reliable and efficient biomarkers on the progress of the SMA treatment [33].

In the present study we developed a novel RT-qPCR-based assay to determine baseline level of FL-SMN and SMNΔ7 mRNA expression in SMA patients and normal individuals. The assay was evaluated on a small group of type II and III SMA patients. A sustained variability in the relative amount both FL-SMN and SMNΔ7 transcripts among these patients was found, which prevents the use of these parameters as biomarkers of SMA treatment. The mRNA levels of FL-SMN and SMNΔ7 in the control subjects overlapped with the values for the SMA patients, which represented similar or slightly lower (for FL-SMN) or slightly higher (for SMNΔ7) values. Our data on the SMN baseline expression levels are similar to the results published by others. For example, in intial studies of *SMN2 *gene expression in SMA patients an overlap between SMN/GAPDH ratios in patients and controls was found [34]. In the study of Simard and co-authors, FL-SMN mRNA and SMNΔ7 mRNA significantly varied in the range of 0.30 - 1.22 and 0.61 - 2.97 [11]. Absolute quantification of FL-SMN mRNA in SMA patients revealed a broad range of variability - 33.75 - 123.00 [12]. The relatively high level of SMNΔ7 mRNA found in our patients compared to controls is an interesting feature that has also been described previously [10,35]. The role of SMNΔ7 mRNA in the development of SMA is unclear. Higher SMNΔ7 mRNA level has been shown to negatively influence the SMA development via neurotoxicity mediated by a pro-apoptotic action of the truncated SMN protein [36]. However, an increased expression of SMNΔ7 mRNA has been suggested to lead to a higher life expectancy in an SMA mouse model [37].

In accordance with the literature, FL-SMN/SMNΔ7 mRNA ratio was used in our analysis in order to avoid confounding effects of FL-SMN and SMNΔ7 mRNA level fluctuations [13]. It can be seen in Figure 3c that the quantification of FL-SMN/SMNΔ7 mRNA ratio revealed a difference between SMA patients and healthy controls. This parameter varied in the range of 0.56 - 2.10 and does not overlap with control values. Thus, we suggest that the FL-SMN/SMNΔ7 mRNA ratio could be used as a diagnostic biomarker for discrimination between SMA patients and normal individuals. It could also be used to determine the effect of the SMA treatment by drugs which are able to correct exon 7 splicing, e.g. histone deacetylation inhibitors. It should be noted that the mechanism of action of some histone deacetylation inhibitors is complex, for example it seems that valproic acid affects both *SMN *gene expression and splicing [38]. However, the clear difference in FL-SMN/SMNΔ7 mRNA ratio between SMA patients and normal individuals, as found in this study and also shown by others, suggests the usefulness of this biomarker for detecting changes during SMA pharmacotherapy.

## Conclusions

The *SMN2 *gene copy quantification in SMA patients can be used as a prognostic tool for discrimination between the SMA type II and SMA type III diagnosis. A novel RT-qPCR-based assay can be used to determine the FL-SMN/SMNΔ7 mRNA ratio. This ratio could be a diagnostic biomarker for monitoring changes during SMA pharmacotherapy.

## Competing interests

The authors declare that they have no competing interests.

## Authors' contributions

All authors read and approved the final manuscript. The contributors are listed in the parentheses: study concept and design (AVK and VGV); acquisition of data (GYZ, AVK, RC and AAE); analysis and interpretation of data (GYZ, AVK and MRA); drafting of the manuscript (GYZ and AVK); critical revision of the manuscript (MRA, HBS and VSB); obtaining of funding (AVK and HBS); clinical support (VGV); study supervision (AVK and VSB).

## Pre-publication history

The pre-publication history for this paper can be accessed here:

http://www.biomedcentral.com/1471-2350/12/96/prepub
